# Relative efficacy of GLP‐1 and GLP‐1/GIP receptor agonists in the prevention of alcohol‐use disorders using a target trial emulation approach

**DOI:** 10.1111/dom.70169

**Published:** 2025-10-07

**Authors:** Alex E. Henney, David R. Riley, Megan Heague, Carl A. Roberts, Theresa J. Hydes, Matthew Anson, David M. Hughes, Uazman Alam, Daniel J. Cuthbertson

**Affiliations:** ^1^ Department of Cardiovascular & Metabolic Medicine University of Liverpool Liverpool UK; ^2^ Metabolism & Nutrition Research Group Liverpool University Hospitals NHS Foundation Trust Merseyside Liverpool UK; ^3^ Liverpool Centre for Cardiovascular Sciences University of Liverpool and Liverpool University Hospitals NHS Foundation Trust Merseyside Liverpool UK; ^4^ Department of Psychology, Institute of Population Health University of Liverpool Merseyside UK; ^5^ Deparment of Health Data Science, Institute of Population Health University of Liverpool Liverpool UK

**Keywords:** cohort study, GLP‐1 analogue, real‐world evidence, type 2 diabetes

## Abstract

**Aims:**

There is growing evidence that the GLP‐1 system is implicated in alcohol and other substance use disorders, and that GLP‐1‐based therapies may have therapeutic relevance in alcohol use disorder (AUD). We aimed to determine the impact of GLP‐1 based therapies on incident AUDs in a real‐world setting in patients with T2D.

**Material and Methods:**

We conducted emulation target trials based on a real‐world network of electronic health records (EHRs) from over 120 million patients in the United States of America. Four target trials were emulated among eligible patients with type 2 diabetes (T2D) who had no prior AUD diagnosis by comparing tirzepatide, semaglutide, liraglutide, and dulaglutide with DPP4 inhibitors (DPP4i). First‐ever diagnosis of AUD occurred within an 18‐month follow‐up period and was examined using Kaplan–Meier survival analyses. Four target trial cohorts were generated and compared with a reference arm of patients treated with DPP4i: cohort (1) treatment with tirzepatide; cohort (2) treatment with semaglutide; cohort (3) treatment with liraglutide; and cohort (4) treatment with dulaglutide. Cohorts underwent propensity score matching 1:1 for confounders. We examined rates of incident AUD (ICD‐10 code F10) and performed head‐to‐head analyses of the incretin‐based therapies. We also performed sensitivity analyses relating to whether treatment was adjunctive therapy with metformin and by treatment adherence.

**Results:**

After propensity‐score matching, we identified four target trials of patients treated with tirzepatide (*n* = 7165), semaglutide (*n* = 20 198), liraglutide (*n* = 6565), and dulaglutide (*n* = 19 061); 1:1 with the reference (DPP4i) patients. Tirzepatide and semaglutide (but not liraglutide or dulaglutide) were associated with significant risk reduction of incident AUD compared to DPP4i (hazard ratio 0.47 [95% confidence interval 0.29, 0.75] and 0.68 [0.52, 0.89], respectively). Head‐to‐head comparison revealed tirzepatide had a significant risk reduction compared to liraglutide in incident AUD (0.47 [0.24, 0.92]).

**Conclusion:**

In patients with T2D, tirzepatide and semaglutide treatment is associated with a lower incidence of AUD; robust randomised, controlled evidence for the use of these drugs for this novel indication is appropriate.

## 1 INTRODUCTION

The management of type 2 diabetes (T2D) and obesity has been revolutionised by glucagon‐like peptide‐1 receptor agonists (GLP‐1 RAs) (e.g., dulaglutide, liraglutide, semaglutide), and, more recently, dual agonists of GLP‐1 and glucose‐dependent insulinotropic polypeptide (GIP) receptors, such as tirzepatide. The applications of these drugs are myriad and include glucose lowering,^1^, ^2^, ^3^, ^4^, ^5^ weight loss, cardiorenal protection,^6^, ^7^, ^8^, ^9^ and treatment of metabolic dysfunction‐associated steatotic liver disease (MASLD)^10^ and obstructive sleep apnoea.^11^ However, alcohol excess and metabolic disease synergistically impact clinical outcomes.^12^, ^13^, ^14^ Patients with MetALD, characterised by hepatic steatosis, metabolic dysfunction, and moderate alcohol intake, have an increased risk of cardiovascular disease and mortality compared to patients with MASLD alone.^15^ Over 100 million cases of alcohol‐use disorders (AUDs) were reported in 2016 globally, the most prevalent of substance misuse disorders, resulting in 99.2 million disability adjusted life years (DALYs).^16^

AUD is a chronic relapsing disorder, resulting in brain adaptations from repeated alcohol use that lead to addiction.^17^, ^18^, ^19^ Due to the heterogeneity of the pathophysiological mechanisms underpinning AUD, there are limited pharmacological options approved as treatment, and there is no high‐grade evidence for pharmacologically controlled drinking in the treatment of AUD.^20^ Given the multifactorial aetiology of AUD and complex neurobiology, there is a pressing need for the development of novel and efficacious pharmacotherapy to treat AUD.

Observations of reduced drinking in people with AUD who are being treated with GLP‐1 and GLP‐1/GIP dual RAs have generated interest in the potential of these medications for treating AUD.^21^ Exenatide may reduce heavy drinking days and total alcohol intake in patients with obesity,^22^ while a cohort study from Denmark, including patients treated with GLP‐1 RAs (not including semaglutide or tirzepatide), demonstrated a 55% reduced risk of alcohol‐related events over 3 months.^23^ A large real‐world study assessing the association between semaglutide and AUD suggested >50% risk reduction in semaglutide treated patients; this analysis did not include tirzepatide.^24^, ^25^ These important findings in humans have been mechanistically evaluated in animal models.^26^

To date, real‐world evidence relating to the impact of tirzepatide and liraglutide is absent, whilst findings of previous real‐world evidence assessing semaglutide may be influenced by residual bias relating to study design and underlying alcohol consumption or liver disease.^27^ The potential clinical implications of a potent drug to treat AUD which can simultaneously improve cardiometabolic health are of huge public health significance and could, for the first time, provide a therapeutic option to the alarming and unaddressed rising tide of liver‐related mortality.^28^ Therefore, the aims of this study were to assess the relative effectiveness of tirzepatide, semaglutide, liraglutide, and dulaglutide in reducing incident AUDs.

## 2 METHODS

### 2.1 Specification of the target trials

#### 2.1.1 Study overview

We compared the new use of tirzepatide, semaglutide, liraglutide, and dulaglutide with the new use of DPP4i on a first‐time diagnosis of AUD using a target trial emulation framework.^29^ DPP4i was selected as the reference arm owing to its lack of pleiotropic effects beyond glycaemic control, consistent with previous methodology. Both DPP4i and GLP‐1/GIP RAs are typically introduced as second‐ or third‐line therapy in patients with T2D.^23^ Supplementary Material lists key protocol components. The target trials are specified as follows (Supplementary Material Table 1).

### 2.2 Eligibility criteria

*Inclusion criteria* for all target trials included patients with T2D who had medical encounters with a Health Care Organisation (HCO) in the United States of America (USA) between May 2022 and November 2023, were prescribed one of tirzepatide, semaglutide, liraglutide, dulaglutide or DPP4i during this time window, and were diagnosed with at least one (metabolic syndrome component, for example, obesity, hypertension, dyslipidaemia, or heart disease, stroke, or HbA1c ≥8.5%) as mandated in GLP‐1 (± GIP) RA prescription guidelines.

*Exclusion criteria* included a diagnosis of type 1 diabetes, history of AUD prior to index event, co‐prescription of any of the treatment or reference medications, contraindications to initiating GLP‐1 (±GIP) RA therapy (pancreatitis, thyroid cancer, gallstones, gastroparesis) based on contraindications, warnings, and limited use information for tirzepatide, semaglutide, liraglutide or dulaglutide, and had no use of any glucose‐lowering therapy within the past 6 months (‘new user’).

#### 2.2.1 Baseline HbA1c

Although some glucose‐lowering agent trials mandate patients have a HbA1c between a given range, we did not apply such criteria. Randomised, controlled trials may mandate an upper limit of 10% for HbA1c with insulin then introduced as escalation therapy. However, given our outcome of interest, AUD, is not influenced by escalation to insulin, and given that we are using real‐world data with less concern regarding metabolic safety, we have not prespecified an upper HbA1c limit.

### 2.3 Treatment strategies

In each of the four target trials, treatment strategies were the initiation of tirzepatide, semaglutide, liraglutide, or dulaglutide at baseline (index event) or the initiation of DPP4i at baseline (index event), but not both. Patients cannot have been co‐prescribed any of the drugs evaluated at any point in the electronic health record history. For all treatment strategies, initiation of use is defined as the first prescription for the drug, consistent with an intention‐to‐treat design. The treatment strategy is assigned at baseline, regardless of medication use adherence, medication switch, or add‐on. The index event followed an active comparator, new user design where the analysis was of new starters of each drug, 1 day after drug initiation. Patients were followed up for 18 months.

### 2.4 Study outcomes

#### 2.4.1 Primary outcome

The primary outcome measure was a first‐time diagnosis of (incident) AUD (International Classification of Diseases, 10th revision [ICD‐10] code F10 for ‘Alcohol‐related disorders’; comprised of alcohol abuse and alcohol dependence) as documented in patient electronic health records (EHRs). Alcohol‐related disorders was our proxy marker for AUDs, in line with previous methodology,^24^ which represent alcohol abuse and alcohol dependence related disease.

#### 2.4.2 Secondary outcomes

All‐cause mortality was used as a secondary outcome.

#### 2.4.3 Falsification outcome

Otitis externa, with no known plausible mechanism for risk reduction in favor of any of the study drugs, was used as a falsification outcome.

### 2.5 Follow up

Each eligible patient was followed from the index event until the occurrence of the outcome, death, loss to follow‐up, or 18 months after the index event, whichever occurred first.

### 2.6 Analysis approach

The causal estimates of interest represent the intention‐to‐treat effect of being assigned to the treatment strategies. Cumulative incidences were estimated using the Kaplan–Meier survival analysis in patients who were propensity‐score matched (1:1 using nearest‐neighbour greedy matching with a calliper of 0.25 times the standard deviation) for baseline covariates. Hazard ratios (HRs) and 95% CIs were calculated. All models are adjusted for confounders at baseline by propensity‐score matching baseline covariates.

## 3 EMULATION OF THE TARGET TRIALS

### 3.1 Study design

We explicitly emulated the target trials described previously using data and built‐in analytic functions on the TriNetX Analytics platform. TriNetX (LLC, Cambridge, MA, USA) is a global federated health research network that has access to both inpatient and outpatient electronic medical records from health care organisations internationally; largely secondary and tertiary care providers in North America and Western Europe. This analysis was conducted exclusively on the US Collaborative Network, which contains data from over 120 million patients across 70 HCOs, with access to diagnoses, procedures, medications, laboratory values, and genomic information worldwide. Data were collected in December 2024. The built‐in analytics within the TriNetX Analytic platform analysed patient‐level data; however, only population‐level results are reported to users. TriNetX data are Health Insurance Portability and Accountability Act (HIPAA) de‐identified and access to protected health information is not allowed. Therefore, there is no risk for protected health information disclosure, and Institutional Review Board review was not required. Further details on the network have been described elsewhere.^30^

Each component of the target trial was emulated using EHRs from the TriNetX Analytics platform. Patients were classified into drug groups—treatment arm (tirzepatide, semaglutide, liraglutide, dulaglutide) or reference arm (DPP4i)—based on the first prescription in the study period (May 2022 to November 2023), which was the baseline or index event. The study period was chosen because tirzepatide was approved by the FDA to treat T2D in May 2022, and approved for weight loss in November 2023. Eligibility criteria and 40 baseline covariates were evaluated at baseline. This included a look‐back period set to ‘anytime’ within the TriNetX Analytical platform. This is capped at a maximum of 20 years, and therefore the earliest date looked back to was 2004; however, the most recent recording of the covariate is used. The treatment and reference arms were separately propensity‐score matched for covariates at the baseline to emulate randomisation. After propensity‐score matching, all groups must have been considered well balanced using a standardised mean difference of <0.1.

### 3.2 Propensity score matching

Cohorts were propensity score matched (PSM), in a 1:1 ratio, for age, sex, ethnicity, smoking, and other lifestyle risk factors (lack of physical activity, inappropriate diet and eating habits, gambling and betting, anti‐social behaviour disorders, sleep disturbance), socioeconomic status (problems relating to education and literacy, employment, housing, and psychosocial circumstances), cardiovascular disease (IHD, PVD, HF, CVA), hypertension, dyslipidaemia, cancer, respiratory disease (chronic obstructive pulmonary disease, bronchiectasis, asthma), gastrointestinal disease (oesophagitis, gastritis, gastro‐oesophageal reflux disease, peptic ulcer disease, inflammatory bowel disease), liver disease (fibrosis and cirrhosis of any cause including alcohol‐related liver disease, autoimmune and viral hepatitis, MASLD), body mass index (BMI), glomerular filtration rate (GFR), HbA1c, liver enzymes (alanine aminotransferase (ALT), aspartate aminotransferase (AST), and gamma glutamyl transferase (GGT)) (as a surrogate marker of alcohol consumption^27^), and medication including other blood glucose‐lowering therapies (insulin, metformin, sulfonylureas, sodium‐glucose cotransporter‐2 inhibitors, thiazolidinediones, and other GLP‐1 RAs) and topiramate. All biochemical and anthropometric variables used in the cohort creation (i.e., HbA1c for BMI) must have been the most recent recorded value prior to the index event; however, we cannot state the exact duration for each patient as we do not have individual‐level data. Definitions for all PSM covariates are presented in Supplementary Material Table 2.

### 3.3 Statistical analysis

Statistical analysis was performed in situ within TriNetX. TriNetX uses the R Survival package v3.2‐3. Additionally, for sensitivity analysis, we performed head‐to‐head analyses of the incretin‐based therapies (tirzepatide vs. (i) semaglutide; (ii) liraglutide; and (iii) dulaglutide), mandated that treatment and reference arm drugs must have been add‐on therapy to metformin, that treatment was adhered to for a minimum of 6 months, and stratified patients by body weight (obesity vs. no obesity (BMI greater than or less than 30 kg/m^2^)), sex (male and female), and age (greater than or less than 60 years). The head‐to‐head analyses were conducted using the same methodological approach as the main analyses, namely an active‐comparator, new‐user design with 1:1 propensity score matching for the full set of baseline covariates, followed by Kaplan–Meier survival analyses and HR estimation. These analyses were implemented within the TriNetX platform as prespecified sensitivity analyses. Finally, we calculated *E*‐values, representing the minimum strength of association on the HR scale that an unmeasured confounder would need to have with both the exposure (treatment arm) and the outcome, conditional on the measured confounders, to explain away the observed association; HR + √[HR×(HR‐1)].^31^ The Strengthening the Reporting of Observational Studies in Epidemiology guidelines were followed in the reporting of this cohort study.^32^

## 4 RESULTS

### 4.1 Overall study population

**FIGURE 1:**
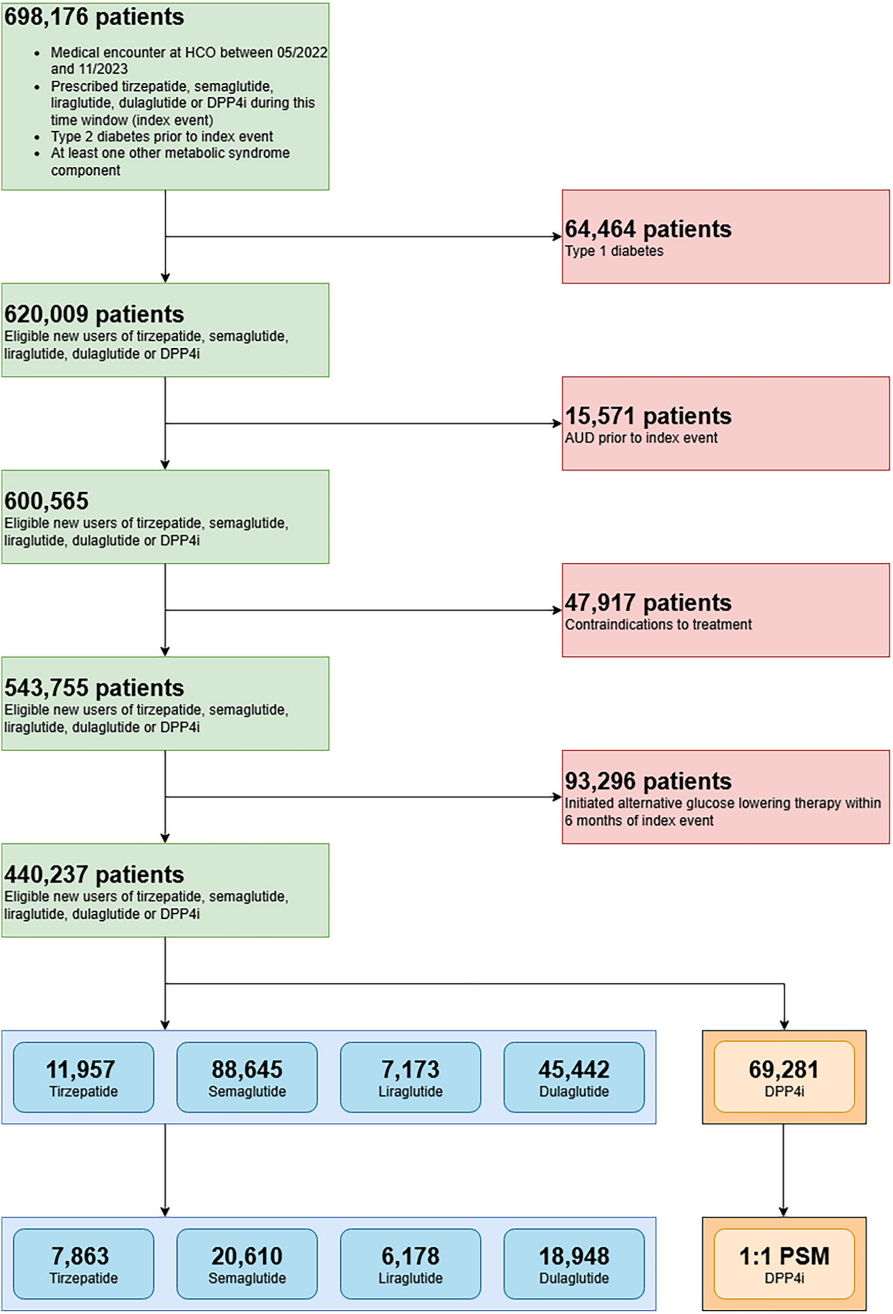
CONSORT diagram demonstrating the eligibility criteria and design of the four target trials comparing tirzepatide, semaglutide, liraglutide, and dulaglutide with DPP4i in reducing incident alcohol‐use disorders in patients with type 2 diabetes. AUD, alcohol‐use disorder; DPP4i, Dipeptidyl peptidase‐4 inhibitor; HCO, Health Care Organisation; USA, United States of America.

A CONSORT diagram demonstrates the cohort composition (Figure 1; with a timeline for the study presented in Supplementary Material Figure 1), including 440 237 new users of the treatment and reference arm drugs, reduced to 11 957 new users of tirzepatide, 88 645 new users of semaglutide, 7173 new users of liraglutide, 45 442 new users of dulaglutide, and 69 281 new users of DPP4i, after excluding patients with co‐prescription of any of the study medications. Baseline characteristics for the tirzepatide and semaglutide target trials are presented in Table 1, whilst those for liraglutide and dulaglutide are presented in Supplementary Material Table 3.

**TABLE 1:** Baseline characteristics of the tirzepatide (vs. DPP4i) and semaglutide (vs. DPP4i) target trial cohorts.

Characteristic	Before propensity score matching			After propensity score matching		
	Treatment	Reference	SMD	Treatment	Reference	SMD
Tirzepatide versus DPP4i						
Demographics						
Numbers (*n*)	11 957	69 281		7863	7863	
Age (years)	55 ± 12	69 ± 12	1.15	59 ± 11	59 ± 13	0.01
Sex, female (%)	56.6	49.0	0.15	53.4	52.6	0.02
Ethnicity, white (%)	67.8	56.5	0.23	64.3	65.1	0.02
Socioeconomic hazards (%)	3.0	3.7	0.04	3.0	3.0	<0.01
Nicotine dependence (%)	10.3	12.8	0.08	11.0	11.5	0.02
Problems related to sleep (%)	0.6	0.4	0.03	0.4	0.4	<0.01
Biochemistry						
HbA1c (%)	7.5 ± 1.8	7.9 ± 1.8	0.22	7.4 ± 1.8	7.5 ± 1.6	0.06
*Data completeness* (%)	75.4	68.3		78.3	74.0	
ALT (U/L)	32 ± 24	24 ± 22	0.33	30 ± 22	30 ± 25	0.04
*Data completeness* (%)	79.9	78.8		75.8	76.2	
AST (U/L)	26 ± 17	23 ± 31	0.10	25 ± 16	25 ± 17	0.02
*Data completeness* (%)	79.6	78.6		75.4	75.9	
GGT (U/L)	71 ± 98	83 ± 159	0.09	80 ± 112	87 ± 156	0.05
*Data completeness* (%)	3.3	3.5		3.2	2.8	
eGFR (mL/min/1.73 m^2^)	83 ± 25	69 ± 29	0.51	79 ± 25	80 ± 28	0.03
*Data completeness* (%)	81.3	82.6		78.1	79.4	
Anthropometrics						
Body mass index (kg/m^2^)	38.8 ± 8.0	30.2 ± 6.7		36.8 ± 7.5	34.9 ± 7.3	
*Data completeness* (%)	74.0	72.3		69.1	68.3	
<30			0.71			0.01
30–35			0.07			0.04
35–40			0.04			0.02
40–45			0.45			0.01
*45–50*			0.40			0.01
*50–55*			0.32			0.03
*55–60*			0.23			0.03
*60–65*			0.15			0.03
*65–70*			0.10			0.03
*>70*			<0.01			<0.01
Comorbidity (%)						
Ischaemic heart disease	13.7	29.3	0.39	16.5	17.1	0.02
Cerebrovascular accident	5.5	16.5	0.36	7.0	6.6	0.02
Peripheral vascular disease	3.3	8.7	0.23	4.0	4.1	<0.01
Heart failure	6.0	15.2	0.30	7.4	7.5	0.01
Hypertension	65.9	83.4	0.42	68.4	69.6	0.03
Cancer	29.1	36.6	0.16	28.4	28.9	0.01
Medication (%)						
Metformin	54.5	69.7	0.32	54.7	55.9	0.02
Insulin	24.0	42.9	0.41	26.7	27.3	0.01
Glipizide	5.8	22.2	0.49	7.4	7.9	0.02
Glimepiride	4.6	14.9	0.36	5.7	5.7	<0.01
Glyburide	1.5	4.9	0.19	1.6	1.6	0.01
Pioglitazone	2.7	8.6	0.26	3.1	3.1	<0.01
Empagliflozin	8.9	13.1	0.13	9.3	9.5	0.01
Dapagliflozin	4.4	5.4	0.04	4.3	4.4	0.01
Canagliflozin	1.6	3.9	0.14	1.8	1.7	0.01
Topiramate	4.6	1.9	0.16	3.4	3.0	0.02
Semaglutide versus DPP4i						
Demographics						
Numbers (*n*)	88 645	69 281		20 610	20 610	
Age (years)	58 ± 13	69 ± 12	0.91	65 ± 11	65 ± 12	0.01
Sex, female (%)	54.2	49.0	0.10	49.9	49.9	<0.01
Ethnicity, white (%)	61.7	56.5	0.11	56.7	57.0	0.01
Socioeconomic hazards (%)	4.1	3.7	0.02	3.6	3.6	<0.01
Nicotine dependence (%)	12.7	12.8	<0.01	12.6	12.6	<0.01
Problems related to sleep (%)	0.7	0.4	0.05	0.4	0.3	0.01
Biochemistry						
HbA1c (%)	7.5 ± 1.8	7.8 ± 1.8	0.18	7.4 ± 1.8	7.5 ± 1.6	0.03
*Data completeness* (%)	70.5	67.5		80.5	74.0	
ALT (U/L)	30 ± 24	24 ± 22	0.25	27 ± 20	26 ± 23	0.02
*Data completeness* (%)	83.1	78.8		71.9	73.4	
AST (U/L)	25 ± 16	23 ± 31	0.06	24 ± 15	24 ± 17	0.02
*Data completeness* (%)	82.8	78.6		71.5	73.1	
GGT (U/L)	66 ± 111	83 ± 159	0.13	65 ± 97	88 ± 168	0.16
*Data completeness* (%)	4.5	3.5		3.8	3.2	
eGFR (mL/min/1.73 m^2^)	81 ± 26	69 ± 29	0.44	73 ± 26	73 ± 29	<0.01
*Data completeness* (%)	84.3	82.6		75.8	77.6	
Anthropometrics						
Body mass index (kg/m^2^)	36.8 ± 8.0	30.2 ± 6.7		32.8 ± 7.1	31.3 ± 6.9	
*Data completeness* (%)	76.7	72.3		68.1	68.0	
*<30*			0.47			<0.01
*30–35*			0.07			0.01
*35–40*			0.39			0.01
*40–45*			0.43			0.03
*45–50*			0.36			0.03
*50–55*			0.28			0.05
*55–60*			0.20			0.03
*60–65*			0.14			0.02
*65–70*			0.09			0.02
*>70*			0.03			<0.01
Comorbidity (%)						
Ischaemic heart disease	18.9	29.3	0.25	23.9	24.1	<0.01
Cerebrovascular accident	8.0	16.5	0.26	12.4	12.4	<0.01
Peripheral vascular disease	4.6	8.7	0.17	6.5	6.6	<0.01
Heart failure	8.7	15.2	0.20	12.0	11.6	0.01
Hypertension	73.9	83.6	0.24	74.5	75.0	0.01
Cancer	35.1	36.6	0.03	31.0	31.5	0.01
Medication (%)						
Metformin	64.1	69.7	0.12	55.7	56.6	0.02
Insulin	31.0	42.9	0.25	34.0	33.8	<0.01
Glipizide	10.9	22.2	0.31	14.5	14.3	0.01
Glimepiride	6.9	14.9	0.26	8.4	8.5	0.01
Glyburide	2.1	4.9	0.15	2.5	2.6	0.01
Pioglitazone	3.8	8.6	0.20	4.4	4.3	<0.01
Empagliflozin	12.0	13.1	0.03	9.2	9.1	<0.01
Dapagliflozin	5.3	5.4	<0.01	3.6	3.6	<0.01
Canagliflozin	2.4	3.9	0.08	1.8	1.8	<0.01
Topiramate	5.1	1.9	0.18	2.2	2.0	0.01

Abbreviations: DPP4i, dipeptidyl peptidase‐4 inhibitor; eGFR, estimated glomerular filtration rate; SMD, standardised mean difference.

### 4.2 Target trial 1: Tirzepatide versus DPP4i

#### 4.2.1 Baseline characteristics

A total of 821 238 patients were identified. 11 957 (14.7%) were prescribed tirzepatide, and 69 281 (85.3%) were prescribed DPP4is. Before matching, the tirzepatide arm was, on average, younger, and more likely to be white and female. They had better glycaemic control and renal function, but higher ALT and AST. They were more likely to be living with obesity, but less likely to have prevalent IHD, CVA, heart failure, peripheral vascular disease, hypertension, and cancer. Finally, they were less commonly co‐prescribed metformin and insulin. After PSM, each cohort was deemed well matched. The total number of participants in each cohort was reduced to 7863 (Table 1). Propensity score density curves are presented in Supplementary Material Figure 2.

#### 4.2.2 Survival analysis

**FIGURE 2:**
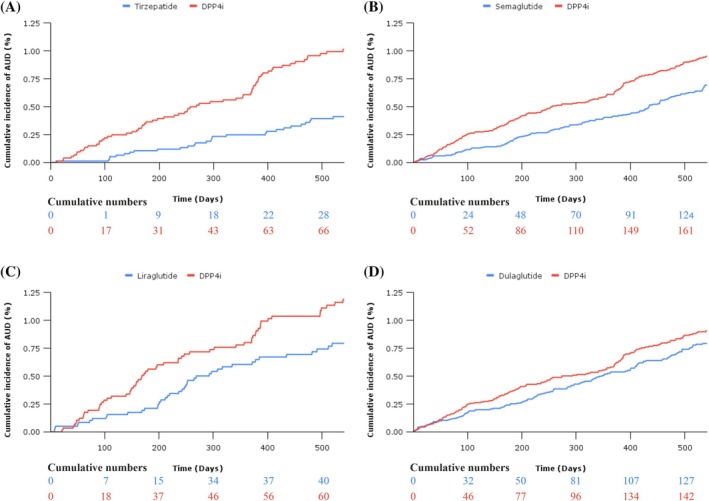
Kaplan–Meier curves demonstrating the efficacy of tirzepatide, semaglutide, liraglutide and dulaglutide (blue), compared to DPP4i (red), in reducing incident alcohol‐use disorders in patients with type 2 diabetes from four target trial emulations. AUD, Alcohol‐use disorders; DPP4i, Dipeptidyl peptidase‐4 inhibitor.

Tirzepatide was associated with a reduced risk of incident AUD (HR 0.40 [95% CI 0.26, 0.62]) (Table 2). Survival curves are presented in Figure 2. The incidence rate in the tirzepatide arm for the first diagnosis of AUD was 3.56 (vs. 8.39 in the DPP4i arm), per 1000 person‐years. Moreover, tirzepatide was associated with a reduced risk of all‐cause mortality (HR 0.28 [95% CI 0.21, 0.37]) (Supplementary Material Figure 3). The mean follow‐up time was 473 days in the tirzepatide arm and 445 days in the DPP4i arm.

**TABLE 2:** Primary and secondary outcomes of alcohol‐use disorders and all‐cause mortality, respectively, according to various pharmacological treatments as part of a series of target trial emulations: GLP1‐RAs/dual GLP‐1/GIP RAs (tirzepatide, semaglutide, and liraglutide) relative to DPP4i (reference).

	Sample size	Outcome (*n*)	5‐year survival probability (%)	Hazard ratio [95% confidence interval]	Log‐Rank test	*p*‐value	*E*‐value
Tirzepatide vs. DPP4i							
*First diagnosis alcohol‐use disorder*							
Reference	7863	66	99.0	Reference			
Tirzepatide	7863	28	99.6	**0.40 [0.26–0.62]**	18.0	<0.01	4.44
*All‐cause mortality*							
Reference	7863	203	96.9	Reference			
Tirzepatide	7863	60	99.1	**0.28 [0.21–0.37]**	86.95	<0.01	6.60
Semaglutide versus DPP4i							
*First diagnosis alcohol‐use disorder*							
Reference	20 610	161	99.0	Reference			
Semaglutide	20 610	124	99.3	**0.71 [0.56–0.90]**	8.29	<0.01	2.17
*All‐cause mortality*							
Reference	20 610	834	95.2	Reference			
Semaglutide	20 610	421	97.7	**0.47 [0.42–0.52]**	170.86	<0.01	3.68
Liraglutide versus DPP4i							
*First diagnosis alcohol‐use disorder*							
Reference	6178	60	98.8	Reference			
Liraglutide	6178	40	99.2	**0.67 [0.45–0.99]**	4.05	0.04	2.35
*All‐cause mortality*							
Reference	6178	257	95.0	Reference			
Liraglutide	6178	221	95.7	0.86 [0.72–1.03]	2.69	0.10	1.00
Dulaglutide versus DPP4i							
*First diagnosis alcohol‐use disorder*							
Reference	18 948	141	99.1	Reference			
Dulaglutide	18 948	127	99.2	0.87 [0.68–1.10]	1.42	0.23	1.00
*All‐cause mortality*							
Reference	18 948	853	94.7	Reference			
Dulaglutide	18 948	585	96.4	**0.66 [0.60–0.74]**	59.57	<0.01	2.40

*Note*: Statistically signifcant results (in bold).

Abbreviation: DPP4i, dipeptidyl peptidase‐4 inhibitor.

#### 4.2.3 Stratified analysis

Tirzepatide was associated with a reduced risk of incident AUD when treatment was adhered to for a minimum of 6 months (HR 0.32 [95% CI 0.17, 0.62]). Tirzepatide reduced incident AUD irrespective of body weight (obesity (HR 0.50 [95% CI 0.28, 0.88]), no obesity (HR 0.24 [95% CI 0.10, 0.58])); however, it was only associated with reduced incident AUD in males (HR 0.59 [95% CI 0.36, 0.97]) and younger adults (HR 0.46 [95% CI 0.25, 0.85]) (Supplementary Material Table 4).

### 4.3 Target trial 2: Semaglutide versus DPP4i

#### 4.3.1 Baseline characteristics

A total of 157 926 patients were identified. 88 645 (56.1%) were prescribed semaglutide, and 69 281 (43.9%) were prescribed DPP4i. Before matching, the semaglutide arm was, on average, younger, and more likely to be female. They had better glycaemic control and renal function, but higher ALT. They were more likely to be living with obesity, but less likely to have prevalent IHD, CVA, heart failure, and hypertension. Finally, they were less likely to be co‐prescribed another glucose‐lowering therapy. After PSM, each cohort was deemed well matched. The total number of participants in each cohort was reduced to 20 610 (Table 1). Propensity score density curves are presented in Supplementary Material Figure 2.

#### 4.3.2 Survival analysis

Semaglutide was associated with a reduced risk of incident AUD (HR 0.71 [95% CI 0.56, 0.90]) (Table 2). Survival curves are presented in Figure 2. The incidence rate in the semaglutide arm for the first diagnosis of AUD was 4.65 (vs. 6.09 in the DPP4i arm) per 1000 person‐years. Moreover, semaglutide was associated with a reduced risk of all‐cause mortality (HR 0.42 [95% CI 0.37, 0.48]) (Supplementary Material Figure 3). The mean follow‐up time was 417 days in the semaglutide arm and 374 days in the DPP4i arm.

#### 4.3.3 Stratified analysis

Semaglutide was associated with a reduced risk of incident AUD when treatment was adhered to for a minimum of 6 months (HR 0.71 [95% CI 0.51, 0.99]). Semaglutide was only associated with reduced incident AUD in patients without obesity (HR 0.62 [95% CI 0.43, 0.89]) and younger adults (HR 0.47 [95% CI 0.29, 0.76]) (Supplementary Material Table 4).

### 4.4 Target trial 3: Liraglutide versus DPP4i

#### 4.4.1 Baseline characteristics

A total of 76,454 patients were identified. 7173 (9.4%) were prescribed liraglutide, and 69,281 (91.6%) were prescribed DPP4is. Before matching, the liraglutide arm was, on average, younger, more likely to be female, and to have adverse socioeconomic status. They had better glycaemic control and renal function, but a higher ALT. They were more likely to be living with obesity but less likely to have prevalent IHD, CVA, and hypertension. Finally, they were more likely to be co‐prescribed insulin. After PSM, each cohort was deemed well matched. The total number of participants in each cohort was reduced to 6178 (Supplementary Material Table 2). Propensity score density curves are presented in Supplementary Material Figure 2.

#### 4.4.2 Survival analysis

Liraglutide was associated with a reduced risk of incident AUD (HR 0.67 [95% CI 0.45, 0.99]) (Table 2). Survival curves are presented in Figure 2. The incidence rate in the liraglutide arm for first diagnosis of AUD was 6.48 (vs. 9.71 in the DPP4i arm) per 1000 person‐years. Liraglutide was not associated with a reduced risk of all‐cause mortality (HR 0.86 [95% CI 0.72, 1.03]) (Supplementary Material Figure 3). The mean follow‐up time was 433 days in the liraglutide arm and 434 days in the DPP4i arm.

#### 4.4.3 Stratified analysis

Liraglutide was only associated with reduced AUD in males (HR 0.47 [95% CI 0.24–0.90]) (Supplementary Material Table 4).

### 4.5 Target trial 4: Dulaglutide versus DPP4i

#### 4.5.1 Baseline characteristics

A total of 114 723 patients were identified. 45 442 (39.6%) were prescribed dulaglutide, and 69 281 (60.4%) were prescribed DPP4is. The dulaglutide arm was, on average, younger and more likely to smoke. They had worse glycaemic control and a higher ALT, but better renal function. They were more likely to be living with obesity, but less likely to have prevalent IHD, CVA, heart failure, and hypertension. Finally, they were more likely to be co‐prescribed insulin. After PSM, each cohort was deemed well matched. The total number of participants in each cohort was reduced to 18 948 (Supplementary Material Table 2). Propensity score density curves are presented in Supplementary Material Figure 2.

#### 4.5.2 Survival analysis

Dulaglutide was not associated with a reduced risk of incident AUD (HR 0.87 [95% CI 0.68–1.10]) (Table 2). Survival curves are presented in Figure 2. The incidence rate in the dulaglutide arm for first diagnosis of AUD was 6.71 (vs. 7.44 in the DPP4i arm) per 1000 person‐years. Dulaglutide was associated with a reduced risk of all‐cause mortality (HR 0.66 [0.60–0.74]) (Supplementary Material Figure 4). The mean follow‐up time was 456 days in the dulaglutide arm and 441 days in the DPP4i arm.

#### 4.5.3 Stratified analysis

Dulaglutide was not associated with reduced incident AUD in ant stratified analysis (Supplementary Material Table 4).

### 4.6 Head‐to‐head analyses

**FIGURE 3:**
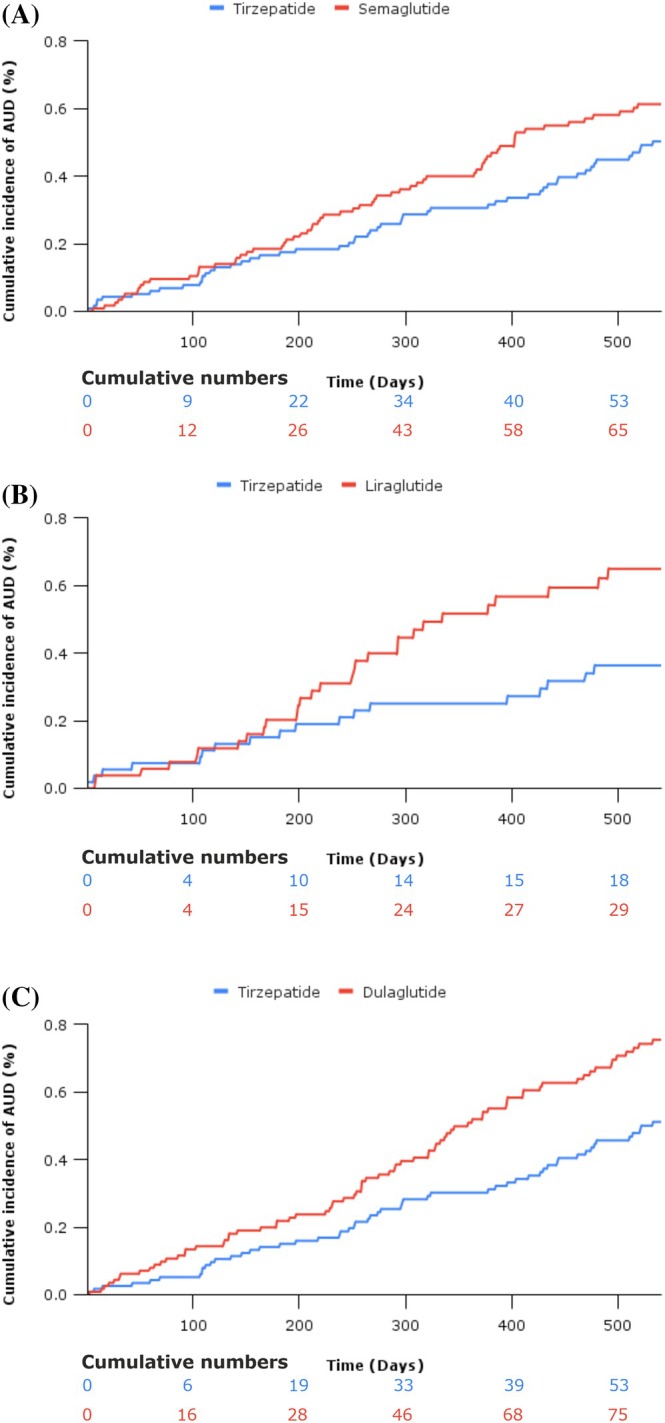
Kaplan–Meier curves demonstrating the head‐to‐head efficacy of tirzepatide, against (a) semaglutide, (b) liraglutide, and (c) dulaglutide in reducing incident alcohol‐use disorders in patients with type 2 diabetes from three target trial emulations. AUD, alcohol‐use disorders.

Tirzepatide was associated with a reduced risk of incident AUD compared to dulaglutide (HR 0.67 [95% CI 0.47, 0.96]), but not liraglutide (HR 0.56 [95% CI 0.31, 1.01]) or semaglutide (HR 0.81 [95% CI 0.56, 1.16]). Results are presented in Supplementary Material Table 4, with survival curves in Figure 3.

## 5 DISCUSSION

This is the first study to assess the effectiveness of four GLP‐1 receptor‐based agonists (tirzepatide, semaglutide, liraglutide, and dulaglutide) against another predominantly glucose‐lowering therapy and provide head‐to‐head analyses, demonstrating a reduction in incident AUDs in patients with type 2 diabetes. We highlight that tirzepatide is associated with the strongest reduction in incident AUDs (60% less), with semaglutide at 29% and liraglutide at 31%, compared to DPP4i. When compared head‐to‐head, tirzepatide was associated with significant risk reductions compared to dulaglutide, whilst survival curves demonstrate clear separation over 18 months of follow‐up when compared against semaglutide and liraglutide despite not reaching statistical significance.

Data on the effects of GLP‐1 RAs on AUDs remain scarce.^21^ Our study shares similarities with animal mechanistic models and human data, including anecdotal case series, real‐world cohort, and clinical trials.^23^, ^24^, ^26^, ^33^, ^34^ The important finding that tirzepatide may be associated with the greatest risk reduction of incident AUDs supports previous smaller observational evidence from patients recruited from a social media platform.^33^ Attribution mapping of social media posts referencing tirzepatide on Reddit, with follow‐up of selected participants, as well as a separate case series, reported decreased symptoms of alcohol excess in patients treated with these incretin‐based therapies.^33^, ^34^ They also reported decreased symptoms of alcohol excess in patients treated with semaglutide, which has also been reflected in a large real‐world evidence study using data from TriNetX.^24^ The effect size of semaglutide on incident AUD reported by Wang et al. was greater than what we produced in our study (55% vs. 29%), although we suggest that this likely related to reduced residual confounding following our TTE design with stricter eligibility criteria (including being geographically well defined), as well as the inclusion of liver enzymes in the PSM model, which was previously omitted. Moreover, less potent (earlier generation) GLP‐1 RAs may only be effective for the first 3 months of treatment, whereas our survival curves demonstrate prolonged effects up to 18 months in more potent incretin‐based therapies such as tirzepatide and semaglutide. Therefore, the results of our study are contradictory to a previous theory proposing the observed reduction in AUDs with GLP‐1 RA treatment related to common associated side effects (including nausea and fatigue) that typically resolve with dose titration over several months.^23^ Finally, RCT data from patients treated with exenatide found a significant reduction in heavy drinking days and total alcohol intake only in patients with obesity.^22^

Although we could not examine the mechanistic interplay between tirzepatide, semaglutide, or liraglutide with reduced AUDs in our study, previous animal models and RCTs provide mechanistic insight. An RCT (using functional MRI) assessing the association between exenatide administration and alcohol intake noted that patients in the treatment arm had attenuated alcohol cue reactivity in the ventral striatum and septal area, whilst dopamine transporter availability was also lower, which taken together suggests reduced incentive salience (or ‘wanting’) of alcohol.^22^ This reduced incentive salience has been corroborated in animal models which suggest that administration of GLP‐1 RAs extinguishes alcohol‐seeking behaviour, and this is potentially mediated by dopaminergic release in the nucleus accumbens.^26^ There is less mechanistic evidence regarding GIP receptor agonism and AUD; however, overlap exists in receptor distribution in the central nervous system between GIP and GLP‐1, whilst similarly GIP receptor agonism appears to be involved in brain regions important for learning and memory (which are both aspects of addictive behaviours); so we could speculate that these two receptor systems work synergistically,^35^ hence tirzepatide being associated with statistically significant superiority over liraglutide, as well as an insignificant beneficial signal over semaglutide and dulaglutide. Moreover, in addition to gut‐brain‐axis signalling, GLP‐1 RA treatments impose anti‐inflammatory effects which may be beneficial in treating substance misuse,^36^ whilst the delayed gastric emptying that occurs following GLP‐1 receptor activation may decrease alcohol absorption, thereby hampering rewarding effects and enhancing the adverse effects of alcohol through conversion to acetaldehyde.^37^ Finally, a further important consideration is that initiation of GLP‐1‐based therapies may itself prompt lifestyle modification. These drugs are typically introduced in the context of intensive diabetes and obesity management, often accompanied by weight loss, dietary adjustments, and healthier behaviours. Such behavioural changes may contribute to the observed reduction in incident AUD, independent of any direct pharmacological effect. Although we attempted to account for baseline comorbidities and liver enzyme levels in propensity‐score matching, residual confounding and misclassification remain possible. Therefore, our findings may reflect both drug‐related mechanisms and indirect benefits mediated through lifestyle modification, reinforcing the need for RCTs to disentangle these effects.

So, what are the clinical implications? Our study provides real‐world evidence of the therapeutic benefit of GLP‐1 and dual GLP‐1/GLP, RAs for AUDs in patients living with T2D. Patients with AUDs often share comorbidities including cardiometabolic disturbance (e.g., hypertension, heart failure, arrhythmias, stroke) and complex psychiatry.^38^, ^39^ Given evidence of accelerated rates of cardiovascular and liver disease, cancer, and all‐cause mortality in patients with concomitant alcohol excess and metabolic syndrome compared to either alone, drugs that improve both risk factors simultaneously have great appeal.^14^ Globally, alcohol use was the seventh leading risk factor for deaths and DALYs in 2016, and the leading cause of death among people aged 15–49 years.^40^ Furthermore, the social and economic costs of alcohol‐related harm are profound. Meta‐analysis of 29 studies identified that the mean costs of alcohol use were equivalent to 1.5% of the GDP.^41^ There is huge potential to impact the substantial harm associated with alcohol excess. Additionally, recent evidence in patients suggests treatment with semaglutide is associated with a lower risk of suicidal ideation,^42^ with alcohol dependence and suicide risk being intertwined. However, randomised controlled trial data are needed for confirmation, with limited other current treatment options available for AUD. For context, our data suggest the number (of patients treated) needed to prevent (NNP) one incident AUD with tirzepatide is 208.^43^ Future research, once data are available, needs to confirm reproducible findings following treatment with tirzepatide in patients with obesity without T2D. Finally, it is important to distinguish between prevention of new‐onset AUD and treatment of established AUD, which are related but distinct clinical questions. Our study specifically examined the incidence of first‐ever AUD diagnoses and therefore cannot provide direct evidence regarding therapeutic efficacy in patients with pre‐existing AUD. While GLP‐1‐based therapies have generated interest as potential treatments for reducing alcohol intake in patients with established disorders, this remains to be demonstrated in robust clinical trials. Coding for recurrent AUD using ICD‐10 classification is weak given heterogeneity in documentation in clinical practice. For example, patients with a prior AUD diagnosis in their medical history will often have this recurrently recorded in clinical notes, regardless of whether they still engage in excess consumption. We therefore felt it inappropriate to use repeat AUD coding as a secondary outcome in this paper. As such, our findings should be interpreted as supporting a potential preventive role, with the possibility of therapeutic benefit remaining an important avenue for future investigation.

We acknowledge limitations to this research. First, these are real‐world data and therefore do not provide randomised or controlled comparisons. In a randomised trial, the interval between the time when an individual first met the eligibility criteria and the time the individual started treatment or control would be the same. However, in our real‐world study, to meet the eligibility criteria, a patient must have been started on one of the respective treatment or control drugs (tirzepatide, semaglutide, liraglutide, dulaglutide, or DPP4i), and therefore it is not possible to say the period of time between which they were theoretically eligible for the treatment and starting the treatment itself. Second, in data extracted from electronic health records in an administrative database, there is potential for a lack of data completeness. This is amplified in the use of open circuit databases like TriNetX where it is possible that a patient may move outside of the HCO and therefore longitudinal data is lost; specifically, this may impact the accuracy of the true incidence rate of AUD, although it would be expected that the number of patients moving outside of the HCO be comparable between arms. TriNetX will exclude missing values from any relevant analysis, but it does not provide imputation or any other statistical technique. Residual bias confounding remains possible despite PSM with potential confounding variables, such as accurate alcohol consumption and smoking, both being poorly coded. To address this, we PSM for liver enzymes at baseline, a biochemical surrogate for alcohol consumption/baseline liver disease severity (although we acknowledge that these can be normal even in patients with advanced liver disease),^27^ as well as PSM for ischaemic heart disease as a further proxy marker of smoking, and further attempted to reduce the risk of unidentified residual confounding through calculation of *E*‐values as a quantitative bias analysis to assist readers in the interpretation of the strength of our results.^31^ We must also acknowledge the under‐reporting of AUD in a real‐world setting owing to potential clinician reluctance to formally diagnose AUD given the associated stigma attached for the patient. The diagnosis of AUD is also heterogenous, with only two of the 11 DSM‐V criteria needed for formal diagnosis. As with any electronic health record‐based study, diagnosis of AUD (or any other outcome) is dependent on the diagnosing clinician. Moreover, the relatively low incidence of AUD in these patients means we may be underpowered to detect treatment differences between GLP‐1 RA head‐to‐head analyses. To assess further effects on other forms of hazardous drinking (i.e., binge drinking), this would require appropriately designed RCTs that specifically look at impacts of drugs in drinking behaviour. As there was not enough follow‐up data available at the time of analysis, we did not examine the effect of tirzepatide, and therefore neither for semaglutide or liraglutide, in patients with obesity. Once this data is available, it would be warranted to assess whether results remain consistent, especially with higher doses used in obesity treatment, of these incretin‐based therapies.

## 6 CONCLUSION

In a large, real‐world setting, in patients with T2D, novel GLP‐1 and dual GLP‐1/GIP receptor agonists are associated with a significantly reduced risk of incident AUDs. These findings have significant public health relevance considering the overlapping and synergistic burden of metabolic disturbance and alcohol excess and require confirmation with randomised controlled trials.

## CONFLICT OF INTEREST STATEMENT

MA receives a fellowship from the Novo Nordisk UK research foundation and JDRF. CAR has acted as a consultant for Boehringer Ingelheim and has received grant funding from Unilever. DJC has received investigator‐initiated grants from Astra Zeneca and Novo Nordisk, support for education from Perspectum, with any financial remuneration from pharmaceutical company consultation made to the University of Liverpool, and serves as the Topic Advisor for Type 2 Diabetes medications for The National Institute for Health and Care Excellence (NICE), UK. GHI is an employee of TriNetX LLC. UA has received honoraria from Procter & Gamble, Viatris, Grunenthal, and Sanofi for educational meetings and funding for attendance at an educational meeting from Daiichi Sankyo. UA has also received investigator‐led funding from Procter & Gamble and is a council member of the Royal Society of Medicine's Vascular, Lipid & Metabolic Medicine Section. All other authors declare that there are no financial relationships or activities that might bias, or be perceived to bias, their contribution to this manuscript.

## Supporting information


**Data S1:** Supporting Information

## Data Availability

This study used population‐level aggregate and de‐identified data collected by the TriNetX Platform, which are available from TriNetX (https://trinetx.com/); however, third‐party restrictions apply to the availability of these data. The data were used under license for this study with restrictions that do not allow for data to be redistributed or made publicly available. To gain access to the data, a request can be made to TriNetX (join@trinetx.com), but costs might be incurred, and a data‐sharing agreement would be necessary. Data specific to this study, including diagnosis codes and group characteristics in aggregated format, are included in the paper as tables, figures, and supplementary files.
