# Supplementary material for: Relative efficacy of GLP‐1 and GLP‐1/GIP receptor agonists in the prevention of alcohol‐use disorders using a target trial emulation approach
Source: Diabetes Obes Metab. 2025 Oct 7;28(1):137–50. doi: 10.1111/dom.70169 (PMC12673470; doi:10.1111/dom.70169)
Supplement: Supplementary file 1 — Data S1: Supporting Information [file DOM-28-137-s001.docx]

**Supplementary Material** Relative efficacy of GLP-1 and GLP-1/GIP receptor agonists in the treatment of alcohol-use disorders using a target trial emulation approach

**Table of contents**

- **Pages 2-5** Protocol
- **Pages 6-8** Target trial specification and emulation table
- **Pages 9-10** Supplementary Material Table 1 (Definitions for all baseline diagnoses, covariates, and outcomes)
- **Page 11** Supplementary Material Figure 1
- **Pages 12-15** Supplementary Material Table 2 (Baseline characteristics of the liraglutide (vs. DPP4i) and dulaglutide (vs. DPP4i) target trial cohorts)
- **Page 16** Supplementary Material Figure 2 (Propensity score density curves for the four target trial emulations)
- **Page 18** Supplementary Figure 4 (Kaplan-Meier curves demonstrating the efficacy of tirzepatide, semaglutide, liraglutide and dulaglutide, compared to DPP4i, in reducing all-cause mortality in patients with type 2 diabetes from four target trial emulations)
- **Page 19-21** Supplementary Material Table 3 (Outcomes of AUD for first diagnosis and recurrence according to various pharmacological treatments)

### **Protocol**

#### **1. Title see amendment**

- Target trial emulations for GLP-1 and GLP-1/GIP receptor agonists in the treatment of alcohol-use disorders

#### **2. Research Question and Objectives**

- **Research Question**: Can treatment with tirzepatide, semaglutide, liraglutide and/or dulaglutide reduce the incidence of major adverse liver outcomes in patients with type 2 diabetes compared to DPP4i?
- **Primary Outcome**: Alcohol-use disorder
- **Secondary Objectives**: All-cause mortality (justification) and otitis externa (falsification)

#### **3. Study Design**

- Target Trial Emulation using retrospective longitudinal data from TriNetX.
- **Study Period**: May 2022 to November 2023 inclusion dates, with 18 months of follow-up from the index event
- **Population**: Adult patients (≥18 years) living with type 2 diabetes

#### **4. Eligibility Criteria**

- **Inclusion Criteria**:
  - Medical encounter with HCO in the USA between May 2022 (approval date of tirzepatide for type 2 diabetes by FDA) and November 2023 (approval date of tirzepatide for obesity by FDA)
  - Prescription of tirzepatide, semaglutide, liraglutide, dulaglutide, or DPP4i during this time with the date of treatment initiation serving as the index event
  - Diagnosis of type 2 diabetes (defined using : **i)** ICD-10 code E11, and/or **ii)** HbA1c >6.4%) on or before the index event
  - Diagnosis of one or more of the metabolic syndrome components (hypertension, obesity, dyslipidaemia) on or before the index event
- **Exclusion Criteria**:
  - Diagnosis of alcohol-use disorder (defined using ICD-10 code F10) on or before the index event
  - Contraindications to treatment with tirzepatide, semaglutide, liraglutide or DPP4i (gallstones, pancreatitis, gastroparesis, thyroid cancer) on or before the index event
  - Diagnosis of type 1 diabetes (defined using ICD-10 code)
  - Initiation on another glucose-lowering therapy in the 6 months preceding the initiation of the treatment or reference drug
  - Co-prescription of the treatment or reference drug

#### **5. Interventions**

- **Treatment Group**: Patients receiving one of **i)** tirzepatide; **ii)** semaglutide; **iii)** liraglutide; **iv)** dulaglutide.
- **Comparison Group**: Patients receiving DPP4i.

#### **6. Assignment of Interventions**

- **Definition of Initiation**: Initiation of **i)** tirzepatide; **ii)** semaglutide; **iii)** liraglutide; **iv)** dulaglutide; **v)** DPP4i, defined as first time the drug is coded on prescription records.
- **Baseline Definition**: The date of initiation of the intervention (or matching initiation date for comparators).

#### **7. Follow-Up**

- **Start of Follow-Up**: Date of intervention/comparator initiation (index event).
- **End of Follow-Up**: Maximum follow-up of 18 months. Patients will be censored if they receive coding for the outcome of interest, patient’s last known fact date, or end of the time-window for analysis

#### **8. Outcomes**

- **Primary Outcome**: Alcohol-use disorder defined using ICD-10 code F10
- **Secondary Outcomes**:
  - All-cause mortality, defined using TriNetX code for ‘deceased’
  - Otitis externa, defined using ICD-10 code H60

#### **9. Data Sources**

- We aim to explicitly emulate the target trials described using data and built-in analytic functions on the TriNetX Analytics platform. TriNetX (LLC, Cambridge, MA, USA) is a global federated health research network that has access to both inpatient and outpatient electronic medical records from health care organisations internationally; largely secondary, and tertiary care providers in North America and Western Europe. This analysis will be conducted using the US Collaborative Network, which contains data from over 120 million patients (from 69 HCOs) with access to diagnoses, procedures, medications, laboratory values and genomic information worldwide. The built-in analytics within the TriNetX Analytic platform will analyse patient-level data; however, only population-level results will be reported to the research team. TriNetX data are HIPAA (Health Insurance Portability and Accountability Act) de-identified and access to protected health information is not allowed. Therefore, there is no risk for protected health information disclosure, and Institutional Review Board review was not required. Further details on the network have been described by Palchuk, M.B., *et al* (A global federated real-world data and analytics platform for research. JAMIA Open, 2023. 6(2): p. ooad035).

#### **10. Confounding and Bias Control**

- **Confounding Variables**: Cohorts will be propensity score matched (PSM), in a 1:1 ratio using greedy nearest neighbour matching, for age, sex, ethnicity, smoking and other lifestyle risk factors (lack of physical activity, inappropriate diet and eating habits, gambling and betting, anti-social behaviour disorders, sleep disturbance), socioeconomic status (problems relating to education and literacy, employment, housing, and psychosocial circumstances), cardiovascular disease (IHD, PVD, HF, CVA), hypertension, dyslipidaemia, cancer, respiratory disease (chronic obstructive pulmonary disease, bronchiectasis, asthma), gastrointestinal disease (oesophagitis, gastritis, gastro-oesophageal reflux disease, peptic ulcer disease, inflammatory bowel disease), liver disease (fibrosis and cirrhosis of any cause including alcohol-related liver disease, autoimmune and viral hepatitis, MASLD), body mass index (BMI), glomerular filtration rate (GFR), HbA1c, liver enzymes (alanine aminotransferase (ALT), aspartate aminotransferase (AST), and gamma glutamyl transferase (GGT)) (as a surrogate marker of alcohol-consumption), and other blood glucose-lowering therapies (insulin, metformin, sulfonylureas, sodium-glucose cotransporter-2 inhibitors, thiazolidinediones, and other GLP-1 RAs).
- **Strategies to Address Confounding**:
  - Propensity score matching
  - Emulation of target trials
- **Handling Missing Data**: TriNetX will perform only complete case-analysis.

#### **11. Statistical Analysis Plan**

- **Analysis Population**: Intention-to-treat, per-protocol
- **Primary Analysis**: Survival analysis with an active comparator new-user model
- **Sensitivity Analyses**: We will perform the following sensitivity analyses:
- Head-to-head analyses of the incretin-based therapies (tirzepatide *vs.* **i)** semaglutide; **ii)** liraglutide; and **iii)** dulaglutide)
- Mandate that treatment and reference arm drugs must have been add-on therapy to metformin
- Mandate that treatment was adhered to for a minimum of 6 months
- Calculate E-values, representing the minimum strength of association on the HR scale that an unmeasured confounder would need to have with both the exposure (treatment arm) and the outcome, conditional on the measured confounders, to explain away the observed association; HR+√[HR×(HR-1)]

#### **12. Assumptions and Limitations**

- Firstly, these will be real-world data and therefore do not provide randomised or controlled comparisons.
- Secondly, in data extracted from electronic health records in an administrative database, there is potential for a lack of data completeness. This is amplified in the use of open circuit databases like TriNetX where it is possible that a patient may move outside of the HCO and therefore longitudinal data is lost. TriNetX will exclude missing values from any relevant analysis, but it does not provide imputation or any other statistical technique.
- Thirdly, residual bias confounding remains possible despite PSM with potential confounding variables, such as accurate alcohol consumption levels at index, and smoking, both being poorly coded. To address this, we will PSM for liver enzymes at baseline as a biochemical surrogate for alcohol consumption/baseline liver disease severity and will further attempt to reduce the risk of unidentified residual confounding through calculation of E-values as a quantitative bias analysis to assist readers in the interpretation of the strength of our results.

#### **13. Ethical Considerations**

- TriNetX data are HIPAA (Health Insurance Portability and Accountability Act) de-identified and access to protected health information is not allowed. Therefore, there is no risk for protected health information disclosure, and Institutional Review Board review was not required

#### **14. Dissemination Plan**

- Plans to publish findings in peer-reviewed journals and present at conferences.

| **Protocol component** | **Target Trial Specification** | **Target Trial Emulation** |
| --- | --- | --- |
| **Eligibility criteria** | ***Inclusions***   - Adult participants, ≥18 years, with T2D (WHO definition: ICD-10 E11 or HbA1c ≥6.5%). - At least one metabolic comorbidity (obesity, hypertension, dyslipidaemia, cardiovascular disease).   ***Exclusions***   - No prior diagnosis of alcohol-use disorder (ICD-10 F10). - No contraindications to GLP-1/GIP RA therapy (e.g., pancreatitis, gallstones, gastroparesis, medullary thyroid cancer). - Excluded if T1D. - Pregnant. - eGFR ≥30 mL/min/1.73m². - HbA1c >10%. | ***Inclusions***   - Adult patients, ≥18 years, in the TriNetX US Collaborative Network with T2D (ICD-10 E11 or HbA1c ≥6.5%) who were prescribed one of tirzepatide, semaglutide, liraglutide, dulaglutide or DPP4i (acting as the placebo). - At least one metabolic syndrome component (obesity, hypertension, dyslipidaemia, or heart disease, stroke, or HbA1c ≥8.5%) as mandated in GLP-1 (+/- GIP) RA prescription guidelines.   ***Exclusions***   - Excluded if AUD (ICD-10 F10) prior to index date. - Excluded if contraindications as per target trial specification. - Excluded if type 1 diabetes (ICD-10 E10). - Excluded if initiated another glucose-lowering therapy within 6 months before index date. - Co-prescription of treatment or reference medications - RCTs may mandate an upper limit of 10% for HbA1c with insulin then introduced as escalation therapy. However, given that we are using real world data with less concern regarding metabolic safety, we have not prespecified an upper HbA1c limit. |
| **Treatment strategies** | Participants were randomised, 1:1, to receive tirzepatide, semaglutide, liraglutide, dulaglutide or blinded placebo. Treatment initiation followed a slow dose escalation regimen every 4 weeks until the maintenance dose was reached. | Initiation of tirzepatide, semaglutide, liraglutide, dulaglutide *vs.* DPP4i (active-comparator, new-user design); with DPP4i acting as the placebo. Index event is the day of treatment prescription. Intention to treat analysis at assignment. Four parallel target trials overall in manuscript. Follow-up up to 18 months. |
| **Treatment assignment** | Central randomization, stratified by age, sex, HbA1c category, and prior therapy use. All participants, investigators, and the sponsor, are blinded. | 1:1 propensity-score matching (nearest-neighbour greedy, caliper 0.1 SD). Balance assessed with SMD (<0.1 threshold in practice). Analyses on matched cohorts. |
| **Outcomes** | ***Primary***   - Incident alcohol-use disorder (AUD) (ICD-10 F10 “alcohol-related disorders”).   ***Secondary***   - All-cause mortality. | Same as target trial.  Outcomes ascertained via ICD-10 codes using the TriNetX platform. |
| **Follow-up** | Baseline = first injection; follow to 40 weeks + 4-week safety or earlier if outcome/discontinuation; rescue allowed per protocol. | Baseline = day after index prescription; follow to first of outcome, death, loss to follow-up, or 18 months. |
| **Causal contrasts** | Intention-to-treat effect (policy estimand).  Per-protocol effect (hypothetical adherence, no switching or rescue therapy). | Observational analogue of ITT using as-treated definition.  Sensitivity analyses: head-to-head GLP-1 RA comparisons; ≥6m adherence requirement; metformin add-on therapy restriction. |
| **Analysis** | Kaplan–Meier survival curves.  Cox proportional hazards model with HRs and 95% CI.  Subgroup analyses by age, sex, comorbidity. | Kaplan–Meier for cumulative incidence; Cox models for HRs with 95% CIs in matched cohorts; TriNetX built-in analytics (R survival v3.2-3). E-values computed for unmeasured confounding; STROBE followed. |
| **Identifying assumptions** | Exchangeability ensured by randomisation.  Positivity (each patient has a chance of being assigned either arm).  Consistency (well-defined interventions). | Exchangeability assumed after PSM on measured confounders.  Positivity ensured by overlap of propensity scores.  • Consistency assumed accurate coding of treatment exposures/outcomes.  Minimal unmeasured confounding (assessed with E-values). |

**Supplementary Material Table 1** Target trial specification and emulation (current observational study). The left column describes the protocol components of the hypothetical randomised controlled trial. The right column describes how each component was emulated using electronic health records from the TriNetX US Collaborative Network to evaluate tirzepatide (semaglutide, liraglutide or dulaglutide) *vs.* DPP4 inhibitors for prevention of incident alcohol-use disorder (AUD). “Causal contrasts” refer to the policy/intention-to-treat estimand (assignment regardless of adherence or rescue therapy) and the hypothetical/per-protocol estimand (if all patients adhered without rescue).

AUD, alcohol use disorder; ITT, intention-to-treat; PSM, propensity score matching; SMD, standardized mean difference; HbA1c, glycated haemoglobin.

| **Diagnosis** | **ICD-10 code** |
| --- | --- |
| **Inclusion criteria** | |
| Type 2 diabetes | E11 (or HbA1c ≥ 6.5%) |
| Obesity | E66.9 (or BMI >30 kg/m^2^) |
| Hypertension | I10 (or blood pressure ≥140/90 mmHg) |
| Dyslipidaemia | E78 (or triglycerides >150 mg/dL) |
| **Exclusion criteria** | |
| Type 1 diabetes | E10 |
| Gastroparesis | K31.84 |
| Thyroid cancer | C73 |
| Gallstones | K80 |
| Acute pancreatitis | K85 |
| Chronic pancreatitis | K86.1 |
| Alcohol-related disorders | F10 |
| **Covariates** | |
| Ischaemic heart disease | I20-I25 |
| Cerebrovascular disease | I60-I69 |
| Peripheral vascular disease | I73 |
| Hypertension | I10 |
| Hypothyroidism | E03 |
| Bronchitis | J40 |
| Emphysema | J43 |
| Bronchiectasis | J47 |
| Asthma | J45 |
| Oesophagitis | K20 |
| Gastro-oesophageal reflux disease | K21 |
| Gastric ulcer | K25 |
| Duodenal ulcer | K26 |
| Crohn’s disease | K50 |
| Ulcerative colitis | K51 |
| Chronic hepatitis | K73 |
| Hepatic failure | K72 |
| Fibrosis or cirrhosis of the liver | K74 |
| Autoimmune hepatitis | K75.4 |
| Cancer | C00-D49 |
| Nicotine dependence | F17.2 |
| Tobacco use | Z72.0 |
| Socioeconomic hazards | Z55-Z65 |
| Inappropriate diet and eating habits | Z72.4 |
| Lack of physical exercise | Z72.3 |
| Gambling and betting | Z72.6 |
| Anti-social behaviour | Z72.81 |
| Problems related to sleep | Z72.82 |
| **Outcome** | |
| Alcohol-related disorders | F10 |

**Supplementary Material Table 2** Definitions for all baseline diagnoses, covariates, and outcomes.


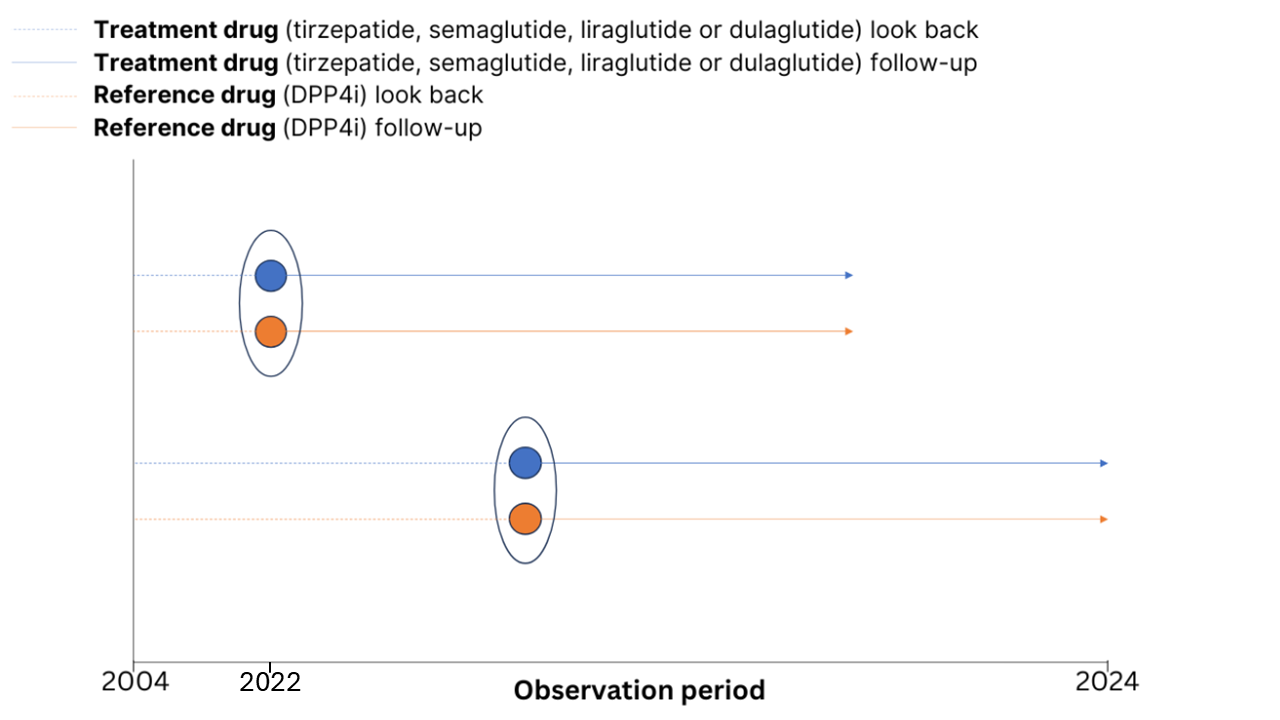


**Supplementary Material Figure 1** Schematic representation of the timeline of the active comparator new user design employed for each target trial. DPP4i, Dipeptidyl peptidase-4 inhibitor. The look back window was set to ‘anytime’ within the TriNetX analytical platform, which is capped at a maximum of 20 years. Therefore, the earliest date of data collection was 2004. The index event was defined as the imitation date of the treatment (tirzepatide, semaglutide, liraglutide or dulaglutide) or reference (DPP4i) drug; an active comparator new user design. The index event must have been between May 2022 and November 2023, and the follow-up was capped at a maximum of 18 months, and therefore the latest possible year for data collection was 2024.

|  | **Before propensity score matching** | | | **After propensity score matching** | | |
| --- | --- | --- | --- | --- | --- | --- |
| Characteristic | Treatment | Reference | SMD | Treatment | Reference | SMD |
| **Liraglutide *vs.* DPP4i** | | | | | | |
| **Demographics** | | | | | | |
| Numbers (n) | 7,173 | 69,281 | | 6,178 | 6,178 | |
| Age (years) | 58 ± 16 | 69 ± 12 | 0.77 | 60 ± 14 | 60 ± 14 | <0.01 |
| Sex, female (%) | 58.1 | 49.0 | 0.18 | 56.0 | 56.2 | <0.01 |
| Ethnicity, white (%) | 57.1 | 56.5 | 0.01 | 57.7 | 58.8 | 0.02 |
| Socioeconomic hazards (%) | 6.1 | 3.7 | 0.11 | 5.5 | 5.4 | 0.01 |
| Nicotine dependence (%) | 14.9 | 12.8 | 0.06 | 14.9 | 15.2 | 0.01 |
| Problems related to sleep (%) | 0.9 | 0.4 | 0.07 | 0.7 | 0.7 | 0.01 |
| **Biochemistry** | | | | | | |
| HbA1c (%) | 7.5 ± 2.0 | 8.0 ± 1.9 | 0.25 | 7.5 ± 2.1 | 7.5 ± 1.6 | 0.25 |
| *Data completeness (%)* | 67.0 | 74.0 | | 66.6 | 67.7 | |
| ALT (U/L) | 28 ± 26 | 24 ± 22 | 0.15 | 27 ± 25 | 28 ± 24 | 0.02 |
| *Data completeness (%)* | 72.1 | 78.8 | | 72.0 | 73.0 | |
| AST (U/L) | 25 ± 22 | 23 ± 31 | 0.06 | 25 ± 22 | 25 ± 18 | <0.01 |
| *Data completeness (%)* | 71.8 | 78.6 | | 71.6 | 72.7 | |
| GGT (U/L) | 61 ± 98 | 83 ± 159 | 0.17 | 66 ± 108 | 97 ± 164 | 0.23 |
| *Data completeness (%)* | 5.0 | 3.5 | | 4.4 | 3.4 | |
| eGFR (mL/min/1.73m2) | 77 ± 31 | 69 ± 29 | 0.28 | 76 ± 30 | 77 ± 32 | 0.04 |
| *Data completeness (%)* | 73.1 | 82.6 | | 74.8 | 77.5 | |
| **Anthropometrics** | | | | | | |
| Body mass index (kg/m^2^) | 36.2 ± 8.6 | 30.2 ± 6.7 | | 35.1 ± 8.1 | 34.1 ± 7.9 | |
| *Data completeness (%)* | 75.4 | 72.3 | | 74.4 | 74.6 | |
| *<30* | | | 0.42 |  | | 0.01 |
| *30-35* | | | 0.04 |  | | 0.04 |
| *35-40* | | | 0.32 |  | | 0.01 |
| *40-45* | | | 0.40 |  | | 0.01 |
| *45-50* | | | 0.37 |  | | 0.02 |
| *50-55* | | | 0.30 |  | | 0.02 |
| *55-60* | | | 0.22 |  | | 0.02 |
| *60-65* | | | 0.18 |  | | 0.01 |
| *65-70* | | | 0.11 |  | | 0.01 |
| *>70* | | | 0.04 |  | | 0.01 |
| **Comorbidity (%)** | | | | | | |
| Ischaemic heart disease | 23.6 | 29.3 | 0.13 | 24.8 | 25.1 | 0.01 |
| Cerebrovascular accident | 11.7 | 16.5 | 0.14 | 12.6 | 12.8 | 0.01 |
| Peripheral vascular disease | 6.9 | 8.7 | 0.07 | 7.0 | 6.7 | 0.02 |
| Heart failure | 13.8 | 15.2 | 0.04 | 14.0 | 14.8 | 0.02 |
| Hypertension | 76.7 | 83.6 | 0.17 | 77.4 | 78.6 | 0.03 |
| Cancer | 34.2 | 36.6 | 0.05 | 34.4 | 35.3 | 0.02 |
| **Medication** | | | | | | |
| Metformin | 65.6 | 69.7 | 0.09 | 63.3 | 64.2 | 0.02 |
| Insulin | 55.3 | 42.9 | 0.25 | 52.5 | 53.6 | 0.02 |
| Glipizide | 14.3 | 22.2 | 0.21 | 15.0 | 15.5 | 0.02 |
| Glimepiride | 8.8 | 14.9 | 0.19 | 9.1 | 9.7 | 0.02 |
| Glyburide | 4.0 | 4.9 | 0.04 | 3.8 | 4.0 | 0.01 |
| Pioglitazone | 5.6 | 8.6 | 0.12 | 5.5 | 6.0 | 0.02 |
| Empagliflozin | 12.6 | 13.1 | 0.01 | 11.7 | 11.9 | 0.01 |
| Dapagliflozin | 4.8 | 5.4 | 0.02 | 4.6 | 4.5 | <0.01 |
| Canagliflozin | 4.0 | 3.9 | 0.01 | 3.4 | 3.3 | 0.01 |
| Topiramate | 6.5 | 1.9 | 0.23 | 4.7 | 4.3 | 0.02 |
| **Dulaglutide *vs.* DPP4i** | | | | | | |
| **Demographics** | | | | | | |
| Numbers (n) | 45,442 | 69,281 | | 18,948 | 18,948 | |
| Age (years) | 59 ± 13 | 69 ± 12 | 0.75 | 65 ± 12 | 65 ± 13 | 0.02 |
| Sex, female (%) | 51.2 | 49.0 | 0.04 | 49.8 | 49.7 | <0.01 |
| Ethnicity, white (%) | 59.3 | 56.5 | 0.06 | 56.8 | 57.3 | 0.01 |
| Socioeconomic hazards (%) | 4.6 | 3.7 | 0.04 | 3.9 | 3.9 | <0.01 |
| Nicotine dependence (%) | 16.3 | 12.8 | 0.10 | 13.6 | 13.8 | <0.01 |
| Problems related to sleep (%) | 0.8 | 0.4 | 0.06 | 0.4 | 0.4 | <0.01 |
| **Biochemistry** GLP-1 | | | | | | |
| HbA1c (%) | 7.9 ±2.0 | 7.5 ± 1.6 | 0.26 | 7.7 ± 1.8 | 7.9 ± 1.8 | 0.13 |
| *Data completeness (%)* | 76.9 | 74.0 | | 69.6 | 69.4 | |
| ALT (U/L) | 29 ± 37 | 24 ± 22 | 0.15 | 28 ± 47 | 26 ± 23 | 0.03 |
| *Data completeness (%)* | 79.3 | 78.8 | | 073.6 | 74.3 | |
| AST (U/L) | 25 ± 21 | 23 ± 31 | 0.04 | 24 ± 17 | 24 ± 18 | 0.01 |
| *Data completeness (%)* | 78.8 | 78.6 | | 73.3 | 73.8 | |
| GGT (U/L) | 78 ± 141 | 83 ± 159 | 0.04 | 72 ± 133 | 88 ± 166 | 0.10 |
| *Data completeness (%)* | 4.0 | 3.5 | | 3.5 | 3.3 | |
| eGFR (mL/min/1.73m2) | 79 ± 28 | 69 ± 29 | 0.35 | 73 ± 28 | 73 ± 30 | 0.01 |
| *Data completeness (%)* | 82.6 | 82.6 | | 77.9 | 78.7 | |
| **Anthropometrics** | | | | | | |
| Body mass index (kg/m^2^) | 35.1 ± 8.0 | 30.2 ± 6.7 | | 32.4 ± 7.1 | 31.4 ± 7.0 | |
| *Data completeness (%)* | 70.7 | 72.3 | | 68.0 | 68.8 | |
| *<30* | | | 0.39 |  | | 0.01 |
| *30-35* | | | 0.02 |  | | 0.01 |
| *35-40* | | | 0.25 |  | | 0.01 |
| *40-45* | | | 0.28 |  | | 0.01 |
| *45-50* | | | 0.24 |  | | 0.02 |
| *50-55* | | | 0.20 |  | | 0.01 |
| *55-60* | | | 0.15 |  | | 0.01 |
| *60-65* | | | 0.10 |  | | 0.01 |
| *65-70* | | | 0.08 |  | | <0.01 |
| *>70* | | | 0.03 |  | | <0.01 |
| **Comorbidity (%)** | | | | | | |
| Ischaemic heart disease | 22.0 | 29.3 | 0.17 | 24.5 | 24.9 | 0.01 |
| Cerebrovascular accident | 10.3 | 16.5 | 0.18 | 13.3 | 13.2 | <0.01 |
| Peripheral vascular disease | 6.3 | 8.7 | 0.09 | 7.2 | 7.1 | <0.01 |
| Heart failure | 11.0 | 15.2 | 0.12 | 12.4 | 12.6 | 0.01 |
| Hypertension | 77.5 | 83.6 | 0.15 | 75.8 | 77.1 | 0.03 |
| Cancer | 33.9 | 36.6 | 0.06 | 32.1 | 32.1 | <0.01 |
| **Medication** | | | | | | |
| Metformin | 72.5 | 69.7 | 0.06 | 60.4 | 61.4 | 0.02 |
| Insulin | 48.5 | 42.9 | 0.11 | 38.3 | 39.1 | 0.02 |
| Glipizide | 18.9 | 22.2 | 0.08 | 16.8 | 16.6 | 0.01 |
| Glimepiride | 10.0 | 14.9 | 0.15 | 9.1 | 9.3 | 0.01 |
| Glyburide | 3.9 | 4.9 | 0.05 | 3.1 | 3.1 | <0.01 |
| Pioglitazone | 6.0 | 8.6 | 0.10 | 5.0 | 4.9 | 0.01 |
| Empagliflozin | 15.8 | 13.1 | 0.08 | 9.9 | 10.0 | <0.01 |
| Dapagliflozin | 6.2 | 5.4 | 0.04 | 4.1 | 3.9 | 0.01 |
| Canagliflozin | 4.0 | 3.9 | <0.01 | 2.2 | 2.0 | 0.01 |
| Topiramate | 4.3 | 1.9 | 0.14 | 2.2 | 2.1 | 0.01 |

**Supplementary Material Table 3** Baseline characteristics of the liraglutide (vs. DPP4i) and dulaglutide (vs. DPP4i) target trial cohorts. SMD, standardised mean difference; DPP4i, Dipeptidyl peptidase-4 inhibitor; eGFR, estimated glomerular filtration rate.


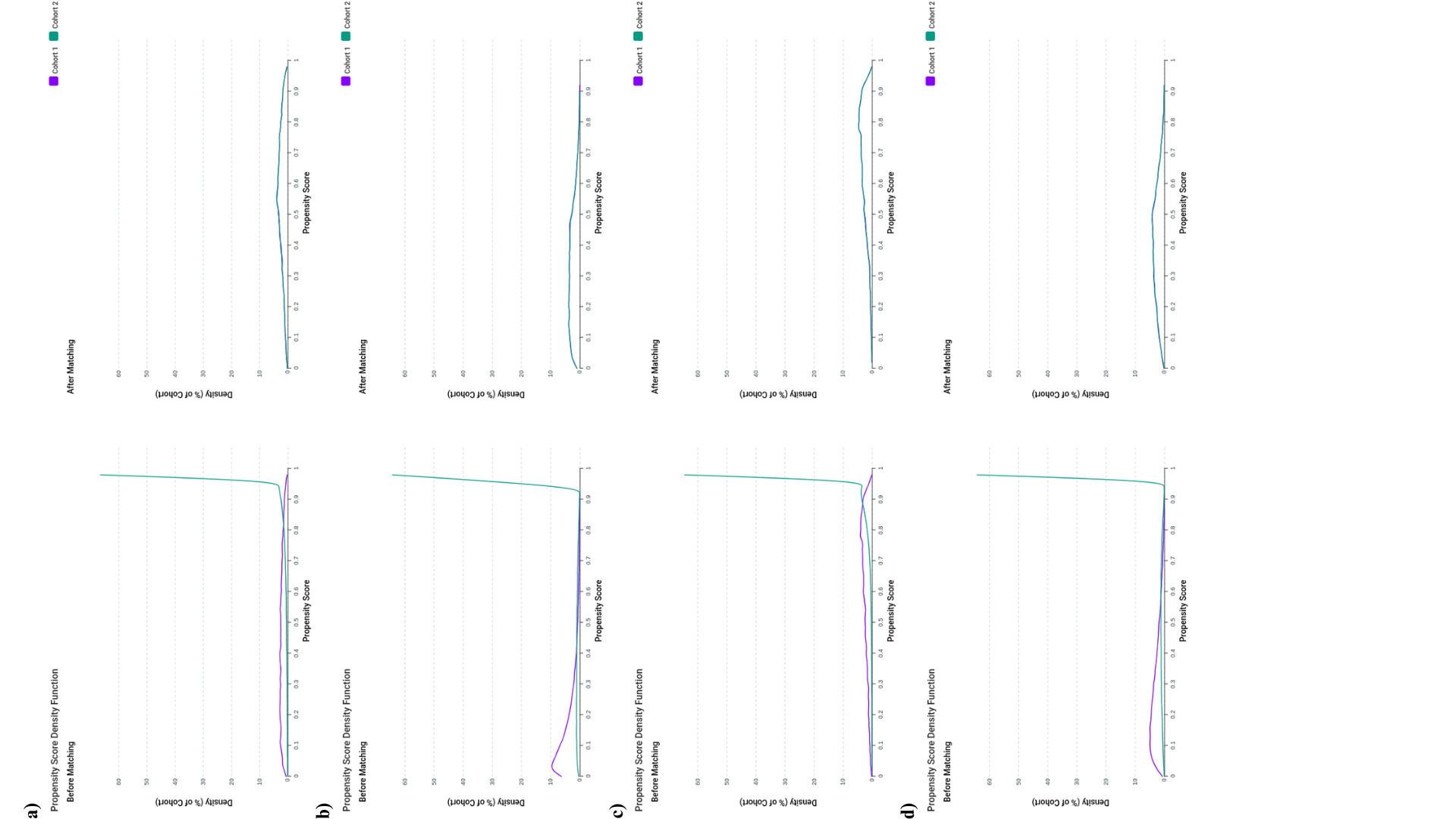


**Supplementary Material Figure 2** Propensity score density curves for the four target trial emulations: **a)** tirzepatide *vs.* DPP4i; **b)** semaglutide *vs.* DPP4i; **c)** liraglutide *vs.* DPP4i; **d)** dulaglutide *vs.* DPP4i.


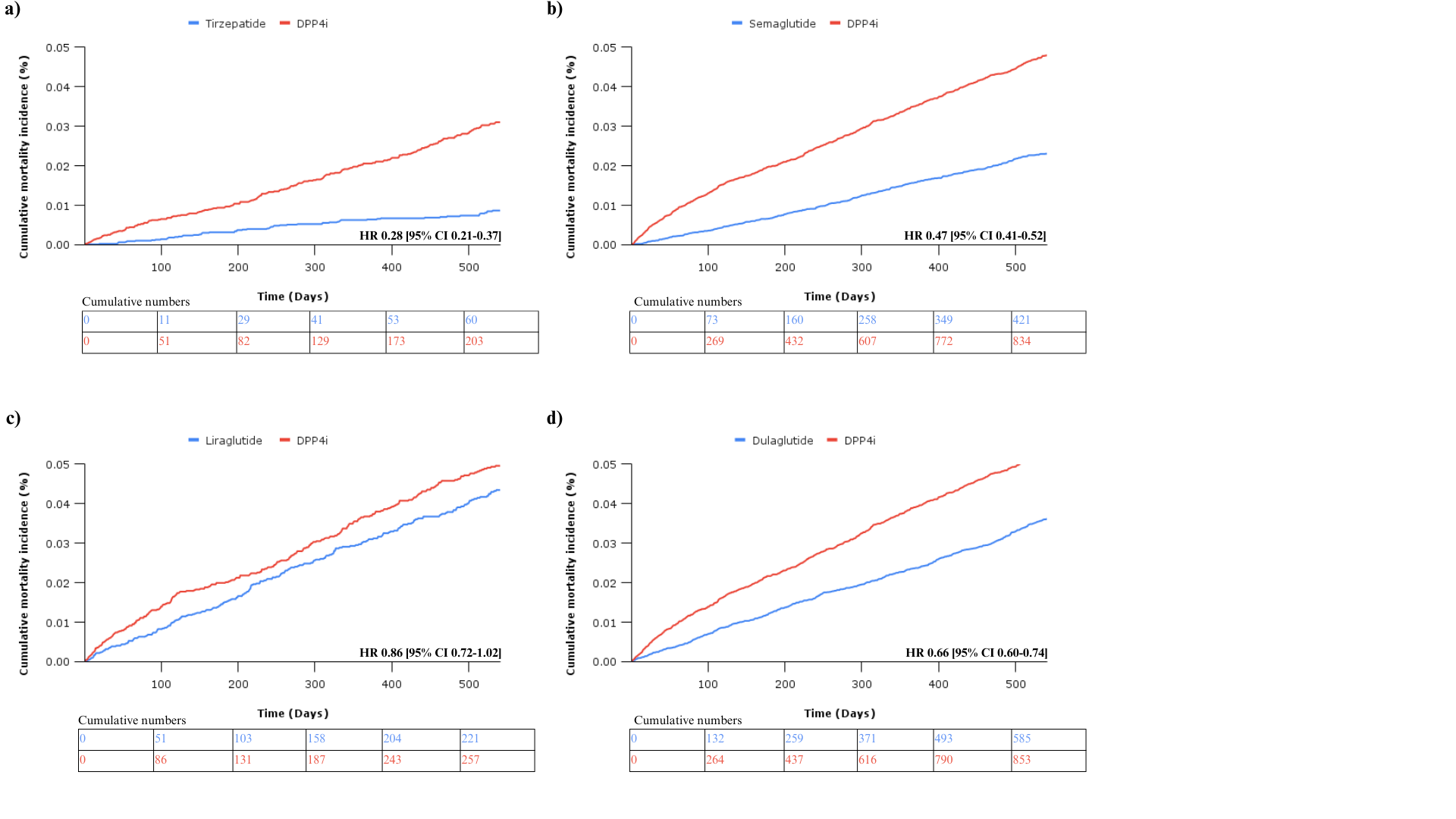


**Supplementary Material Figure 3** Kaplan-Meier curves demonstrating the efficacy of tirzepatide, semaglutide, liraglutide and dulaglutide (blue), compared to DPP4i (red), in reducing all-cause mortality in patients with type 2 diabetes from four target trial emulations. DPP4i, Dipeptidyl peptidase-4 inhibitor.

|  | **Sample size** | **Outcome (n)** | **5-year survival probability (%)** | **Hazard ratio (95% confidence interval)** | **Log-Rank test** | **P value** | **E-value** |
| --- | --- | --- | --- | --- | --- | --- | --- |
| **Tirzepatide *vs.* semaglutide** | | | | | | | |
| ***First diagnosis alcohol-use disorder*** | | | | | | | |
| Semaglutide | 11,814 | 65 | 99.4 | Reference | | | |
| Tirzepatide | 11,814 | 53 | 99.5 | 0.81 [0.56-1.16] | 1.32 | 0.25 | 1.00 |
| ***All-cause mortality*** | | | | | | | |
| Semaglutide | 11,814 | 102 | 99.0 | Reference | | | |
| Tirzepatide | 11,814 | 76 | 99.3 | **0.74 [0.55-0.99]** | 4.00 | 0.04 | 2.00 |
| **Tirzepatide *vs.* liraglutide** | | | | | | | |
| ***First diagnosis alcohol-use disorder*** | | | | | | | |
| Liraglutide | 5,459 | 29 | 99.4 | Reference | | | |
| Tirzepatide | 5,459 | 18 | 99.6 | 0.56 [0.31-1.01] | 3.76 | 0.05 | 1.00 |
| ***All-cause mortality*** | | | | | | | |
| Liraglutide | 5,459 | 152 | 96.6 | Reference | | | |
| Tirzepatide | 5,459 | 51 | 98.9 | **0.30 [0.22-0.41]** | 62.13 | <0.01 | 6.10 |
| **Tirzepatide *vs.* dulaglutide** | | | | | | | |
| ***First diagnosis alcohol-use disorder*** | | | | | | | |
| Dulaglutide | 11,705 | 75 | 99.2 | Reference | | | |
| Tirzepatide | 11,705 | 53 | 99.5 | **0.67 [0.47-0.96]** | 4.99 | 0.03 | 2.35 |
| ***All-cause mortality*** | | | | | | | |
| Dulaglutide | 11,705 | 160 | 98.4 | Reference | | | |
| Tirzepatide | 11,705 | 76 | 99.3 | **0.45 [0.34-0.59]** | 34.21 | <0.01 | 3.90 |

**Supplementary Material Table 3** Outcomes of AUD for first diagnosis and recurrence according to various pharmacological treatments: GLP1-RAs/dual GLP-1/GIP RAs (tirzepatide, semaglutide and liraglutide) relative to DPP4i (reference)

| **Stratified analysis** | **Hazard ratio** | **95% confidence interval** |
| --- | --- | --- |
| ***Tirzepatide vs. DPP4i*** | | |
| ***Obesity*** | | |
| Obesity | **0.50** | **0.28-0.88** |
| No obesity | **0.24** | **0.10-0.58** |
| ***Sex*** | | |
| Male | **0.59** | **0.36-0.97** |
| Female | 0.48 | 0.22-1.03 |
| ***Age*** | | |
| Older | 0.66 | 0.38-1.15 |
| Younger | **0.46** | **0.25-0.85** |
| ***Treatment adherence*** | | |
| Adhered to treatment for at least 6 months | **0.32** | **0.17-0.62** |
| ***Add on therapy*** | | |
| Tirzepatide used as adjunct to metformin | 0.59 | 0.34-1.02 |
| ***Semaglutide vs. DPP4i*** | | |
| ***Obesity*** | | |
| Obesity | 0.70 | 0.45-1.02 |
| No obesity | **0.62** | **0.43-0.89** |
| ***Sex*** | | |
| Male | 0.82 | 0.62-1.10 |
| Female | 0.73 | 0.46-1.14 |
| ***Age*** | | |
| Older | 0.82 | 0.62-1.08 |
| Younger | **0.47** | **0.29-0.76** |
| ***Treatment adherence*** | | |
| Adhered to treatment for at least 6 months | **0.71** | **0.51-0.99** |
| ***Add on therapy*** | | |
| Tirzepatide used as adjunct to metformin | 0.78 | 0.59-1.04 |
| ***Liraglutide vs. DPP4i*** | | |
| ***Obesity*** | | |
| Obesity | 0.78 | 0.46-1.40 |
| No obesity | 0.36 | 0.13-1.01 |
| ***Sex*** | | |
| Male | **0.47** | **0.24-0.90** |
| Female | 0.75 | 0.37-1.52 |
| ***Age*** | | |
| Older | 0.56 | 0.31-1.03 |
| Younger | 0.60 | 0.29-1.26 |
| ***Treatment adherence*** | | |
| Adhered to treatment for at least 6 months | 0.90 | 0.54-1.50 |
| ***Add on therapy*** | | |
| Tirzepatide used as adjunct to metformin | 0.80 | 0.48-1.34 |
| ***Dulaglutide vs. DPP4i*** | | |
| ***Obesity*** | | |
| Obesity | 0.82 | 0.54-1.24 |
| No obesity | 0.79 | 0.55-1.15 |
| ***Sex*** | | |
| Male | 0.84 | 0.63-1.12 |
| Female | 0.70 | 0.44-1.10 |
| ***Age*** | | |
| Older | 0.94 | 0.70-1.27 |
| Younger | 0.72 | 0.47-1.10 |
| ***Treatment adherence*** | | |
| Adhered to treatment for at least 6 months | 0.83 | 0.60-1.15 |
| ***Add on therapy*** | | |
| Tirzepatide used as adjunct to metformin | 0.97 | 0.73-1.27 |

**Supplementary Material Table 4** Outcomes of the stratified analyses from the various target trial emulations.
